# Pterostilbene pre-treatment reduces LPS-induced acute lung injury through activating NR4A1

**DOI:** 10.1080/13880209.2022.2034893

**Published:** 2022-03-10

**Authors:** Ying Li, Shu-Min Wang, Xing Li, Chang-Jun Lv, Ling-Yun Peng, Xiao-Feng Yu, Ying-Jian Song, Cong-Jie Wang

**Affiliations:** a Department of Emergency, Yantai Yuhuangding Hospital, Yantai, Shandong, China; b Department of Station Intergrate Service, Yantai Central Blood, Yantai, Shandong, China; c Binzhou Medical University, Yantai, Shandong, China; d Department of Thoracic Surgery, Yantai Yuhuangding Hospital, Yantai, Shandong, China; e Pulmonary and Critical Care Medicine, Yantai Yuhuangding Hospital, Yantai, Shandong, China

**Keywords:** Lipopolysaccharide-induced, nuclear factor-κB pathway, inflammatory cells, inflammatory factors

## Abstract

**Context:**

Pterostilbene (PTE), a common polyphenol compound, exerts an anti-inflammatory effect in many diseases, including acute lung injury (ALI).

**Objective:**

This study explores the potential mechanism of PTE pre-treatment against lipopolysaccharide (LPS)-induced ALI.

**Materials and methods:**

Sixty Sprague-Dawley rats were divided into control, ALI, 10 mg/kg PTE + LPS, 20 mg/kg PTE + LPS, and 40 mg/kg PTE + LPS groups. At 24 h before LPS instillation, PTE was administered orally. At 2 h before LPS instillation, PTE was again administered orally. After 24 h of LPS treatment, the rats were euthanized. The levels of inflammatory cells and inflammatory factors in the bronchoalveolar lavage fluid (BALF), the expression of nuclear receptor subfamily 4 group A member 1 (NR4A1), and the nuclear factor (NF)-κB pathway-related protein levels were detected. NR4A1 agonist was used to further investigate the mechanism of PTE pre-treatment.

**Results:**

After PTE pre-treatment, the LPS induced inflammation was controlled and the survival rate was increased to 100% from 70% after LPS treatment 24 h. For lung injury score, it decreased to 1.5 from 3.5 after treating 40 mg/kg PTE. Compared with the control group, the expression of NR4A1 in the ALI group was decreased by 20–40%. However, the 40 mg/kg PTE pre-treatment increased the NR4A1 expression by 20–40% in the lung tissue. The results obtained with pre-treatment NR4A1 agonist were similar to those obtained by pre-treatment 40 mg/kg PTE.

**Conclusions:**

PTE pre-treatment might represent an appropriate therapeutic target and strategy for preventing ALI induced by LPS.

## Introduction

Acute lung injury (ALI) is a life-threatening condition characterized by high incidence and mortality rates (Borges et al. 2019). Traumatic injuries may trigger the occurrence of ALI, and they are grossly divided into two categories: direct and indirect injury (Chimenti et al. 2020). As a multifactorial disease, ALI is highly correlated with immune response and inflammatory process (Maeda et al. 2020). Based on previous findings (Fair and Tor 2014), the main cause of ALI appears to be Gram-negative bacterial infection. As the major Gram-negative bacterial component, lipopolysaccharide (LPS) is an important stimulus for the immune response (Nakazato et al. 2018), causing inflammatory leukocyte infiltration and severe lung injury. Previous studies also have introduced LPS-induced ALI animal models to explore more effective methods for overcoming ALI (Eiznhamer et al. 2004; Hu et al. 2017; An et al. 2019). As a serious disease associated with several causative factors (Johnson and Matthay 2010), effective therapeutic strategies for ALI are urgently needed. Therefore, it is crucial to explore more effective methods for the enhancement of immune response against ALI.

Pterostilbene (*trans*-3,5-dimethoxy-4-hydroxystilbene, PTE) is a common polyphenol compound that exists in certain plant foods, such as nuts and berries, and is particularly abundant in blueberries (Mccormack and Mcfadden 2013). PTE was found to be pharmacological safe because it showed no organ-specific or systemic toxicity, including tissue histopathologic examination and regular haematology and clinical chemistry data (Obrador et al. 2021). A previous study reported that the protective effects of PTE against certain diseases, such as in attenuation of vascular disease, may be based on its antioxidant activity (Park et al. 2018). Another previous study also revealed that pre-treatment PTE exerts an anti-inflammatory effect in chronic liver-related diseases (Chen et al. 2017). Recently, researchers have found PTE exerts protective effects on sepsis-induced ALI in a rat model *via* the JAK2/STAT3 pathway (Xue and Li 2020). Interestingly, PTE pre-treatment could inhibit the inflammatory response and oxidative stress for LPS induced ALI by inhibiting nuclear factor (NF)-κB and activating the Nrf2/HO-1 pathway (Zhang et al. 2020). However, the underlying mechanisms involved in the protective effects of PTE on preventing ALI are not completely clear.

The nuclear receptor subfamily 4 group A member (NR4A) family, which consists of three members, NR4A1, NR4A2, and NR4A3, is associated with various cellular processes, including cellular proliferation, apoptosis, and energy utilization (Herring et al. 2019). Herring et al. (2019) also reported that NR4A1 and NR4A2 inhibited cell proliferation in the liver, while NR4A3 had the opposite effect in controlling the proliferation of hepatocytes and hepatic stellate cells. Banno et al. (2019) reported that NR4A1 played a pivotal role in regulating inflammatory responses through its function in the NF-κB pathway. In particular, NR4A1 could upregulate the expression of p65 downstream genes by weakening the binding ability of p65 and DNA. NR4A1 directly promoted the expression of IκB (Huang et al. 2016) and regulated the activity of the NF-κB pathway through non-protein-protein interactions (Kalogeris et al. 2012). When investigating other inflammation-related diseases, NR4A1 was demonstrated to inhibit the inflammatory response and delay disease progression (Nakazato et al. 2018). Therefore, we put forward a hypothesis that NR4A1 plays an important role in the LPS induced ALI, and the pre-protective effects of PTE might be associated with the NR4A1 expression.

In this study, we investigate the effects of PTE pre-treatment on the NR4A1 expression in the LPS-induced ALI and elucidate its potential underlying mechanisms.

## Materials and methods

### Animals

A total of 60 Sprague-Dawley male rats, weighing 200–220 g, were purchased from Jinan Peng Yue Laboratory Animal Technology [SCXK (Shandong) 20190003]. All animals were housed at 23 ± 2 °C, with 55 ± 5% humidity and a 12 h light/dark cycle, and were provided access to food and water *ad libitum*. The study has been approved by the Institutional Animal Care and Use Committee of Yantai Yuhuangding Hospital (Approval No. 2019382). All experiments were followed the Guide for the Care and Use of Laboratory Animals published by the US National Institutes of Health (2011).

### LPS induced ALI model

As reported in a previous study (Liang et al. 2019), an LPS-induced ALI rat model was established. Briefly, rats were anaesthetized with 2% sodium pentobarbital (45 mg/kg), then instilled with *Escherichia coli* LPS (O55:B5, cat. no. 19660, Cayman Chemical Company) at a dosage of 2.5 mg/kg (50 µL) by endotracheal intubation 4–5 times to ensure that all instilled solutions reached the distal lung.

### Animal groups

Different concentrations of PTE in the solvent agent-sunflower oil were prepared and the experimental animals were randomized into five groups, with 10 rats in each group. (i) Control group (Control): At 24 and 2 h before instillation, the solvent agent-sunflower oil (Perecko et al. 2013) was given by oral administration. Then rats were instilled with 50 µL physiological saline by endotracheal intubation 4–5 times. (ii) ALI model group (ALI): At 24 and 2 h before instillation, the solvent agent-sunflower oil was administered orally (Perecko et al. 2013). Then, the rats were instilled with 50 µL *Escherichia coli* LPS (2.5 mg/kg) by endotracheal intubation 4–5 times. (iii) Low dose of PTE group (L-PTE + LPS): At 24 h before LPS instillation, 10 mg/kg PTE (cat. no. orb322752, Biorbyt) was administered orally (Perecko et al. 2013). At 2 h before LPS instillation, 10 mg/kg PTE was again administered orally. (iv) Medium dose of PTE group (M-PTE + LPS): At 24 h before LPS instillation, 20 mg/kg PTE (Perecko et al. 2013) was administered orally. At 2 h before LPS instillation, 20 mg/kg PTE was again administered orally. (v) High dose of PTE group (H-PTE + LPS): At 24 h before LPS instillation, 40 mg/kg PTE (Perecko et al. 2013) was administered orally. At 2 h before LPS instillation, 40 mg/kg PTE was again administered orally.

After 24 h of LPS treatment, the rats were euthanized with an overdose of sodium pentobarbital (150 mg/kg) by intraperitoneal injection. Subsequently, the lung tissue samples and bronchoalveolar lavage fluids (BALFs) were collected for subsequent experiments.

**Figure 1.**
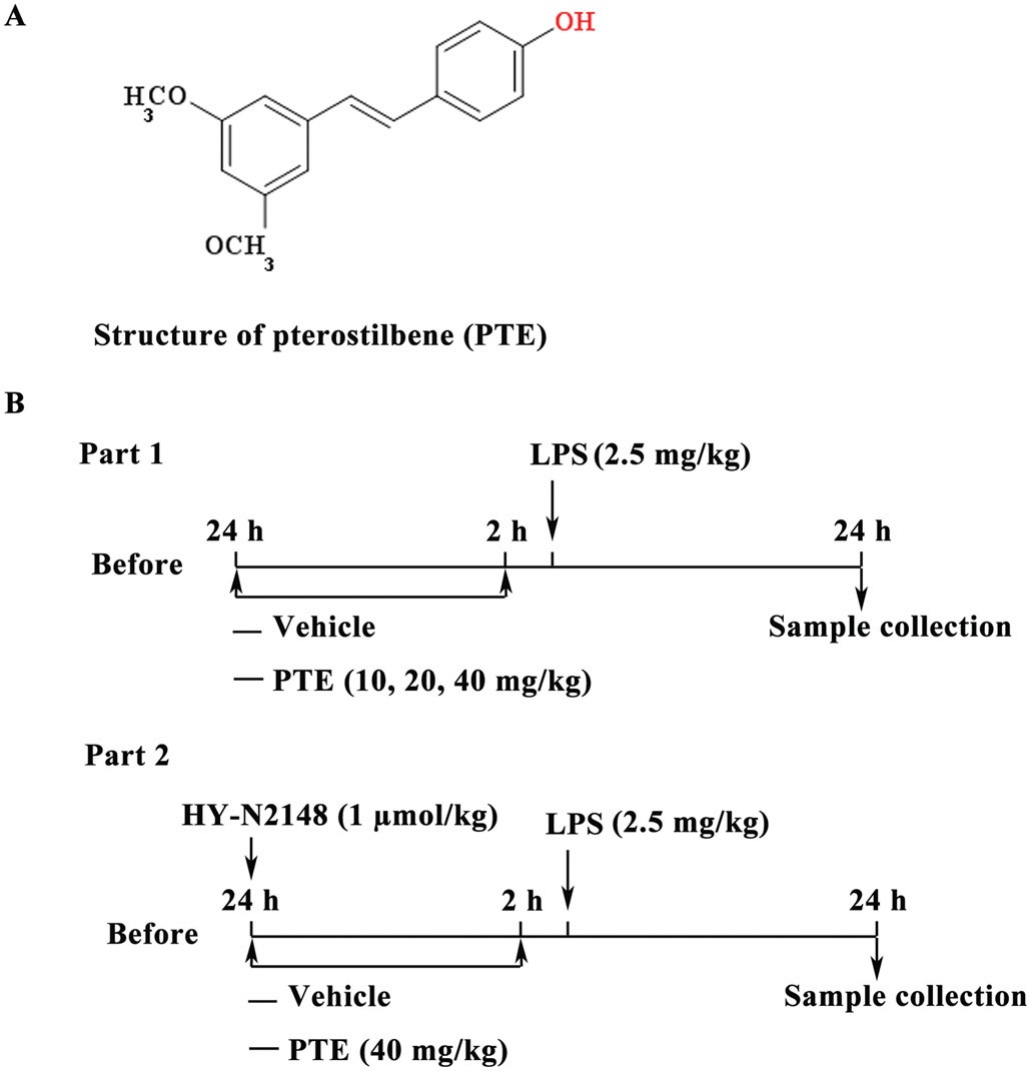
Experimental flow chart. (A) Chemical structure of PTE; (B) The experimental scheme of this study.

To confirm pre-administration of PTE could regulate NR4A1 expression to improve LPS-induced ALI, the NR4A1 agonist (Cytosporone B, cat. no. HY-N2148, MedChemExpress) was used as a control. Specifically, at 24 h before LPS instillation, 50 µL Cytosporone B (1 µM) was intraperitoneally injected in the rats (Xiong et al. 2020). The experimental process is shown in Figure 1. The survival rate was tracked every 6 h after the LPS instillation.

### Haematoxylin-eosin (HE) staining

The lung specimens were fixed with 4% paraformaldehyde [cat. no. M13405, Meryer (Shanghai) Chemical Technology Co., Ltd.] for 24 h, and embedded in paraffin. The specimens (5 µm) were dewaxed with xylene, dehydrated through an ethanol gradient (100% ethanol, 5 min; 95% ethanol, 2 min; 80% ethanol, 2 min; and 70% ethanol, 2 min), and then stained with HE (cat. no. G1120, Beijing Solarbio Science & Technology Co., Ltd.) for 15 min. After 30 s differentiations, the specimens were soaked in warm water at 50 °C for 5 min and stained with eosin for 40 s. After washing, the samples were dehydrated with ethanol and purified with xylene. A researcher who was blinded to the experimental grouping assessed and scored the degree of lung injury on a 0–4 point scale (Jia et al. 2019) as follows: No damage = 0 points; 25% visual field damage = 1 point; 50% visual field damage = 2 points; 75% visual field damage = 3 points; and systemic damage = 4 points.

### Lung wet/dry (W/D) weight ratio

The W/D weight ratio was calculated to determine the water proportion of the lung tissues. The right lung was removed from the chest cavity and weighed. The dry weight was measured after drying the lung at 80 °C for 48 h. Then, the W/D weight ratio we calculated to evaluate tissue edoema.

### Inflammation cells counts

The total cells, neutrophils and macrophages in bronchoalveolar lavage fluids (BALFs) of rats in each group were counted by cell counting plate under an optical microscope (300 cells per smear).

### ELISA

After the rats were euthanized and the thoracic cavity was exposed, 1 mL pre-cooled PBS was carefully injected into the trachea for lavage, and then the liquid was slowly drawn out. This step was repeated three times for each rat and then the liquid was centrifuged at 4 °C (400 *g*, 15 min) to collect the supernatant. ELISA kits were used to measure the levels of IL-1β (cat. no. Z02978-1, GenScript), IL-18 (cat. no. Z03025-10, GenScript), and IL-6 (cat. no. CG39, Novoprotein) in BALFs.

### Immunohistochemistry

The lung samples were fixed with 4% paraformaldehyde for 24 h, embedded with paraffin, and cut into 5 µm sections. After dewaxing with xylene, the specimens were dehydrated through an ethanol gradient. After adding 3% H_2_O_2_ methanol solution for 12 min and citrate buffer at 95 °C for 10 min, the specimens were sealed with 5% bovine serum albumin (BSA; cat. no. A8010, Beijing Solarbio Science & Technology Co., Ltd.) at room temperature for 20 min. The specimens were incubated with primary rabbit anti-human NR4A1 polyclonal antibody (1:100, cat. no. orb127604, Biorbyt) at 4 °C overnight. After washing with PBS, the specimens were incubated with horseradish peroxidase-conjugated goat anti-rabbit IgG (1:800, cat. no. K0034G-AF594, Beijing Solarbio Science & Technology Co., Ltd.) at room temperature for 60 min, then coloured with DAB (cat. no. SW1020, Beijing Solarbio Science & Technology Co., Ltd.) for 10 min and haematoxylin for 2 min, followed by hydrochloric acid alcohol differentiation for 2 s. Finally, the samples were dehydrated through an ethanol gradient, transparentized with xylene, and sealed with neutral resin. The samples were all observed under an optical microscope (Olympus Corporation) at a magnification of 200×. The same area on the slide was selected and analyzed using ImageJ software (version 6; National Institutes of Health).

### Western blot

The lung tissues were dissolved in a cold RIPA buffer (cat. no. R0010, Beijing Solarbio Science & Technology Co., Ltd.) and centrifuged at 12,000 *g* for 25 min at 4 °C. A total protein extraction kit (cat. no. BC3640-50T, Beijing Solarbio Science & Technology Co., Ltd.) was used to extract total protein from samples. Proteins (40 μg per lane) were separated by 10% SDS-PAGE (Bio-Rad Laboratories, Inc.) and then transferred to PVDF membranes (EMD Millipore); the membranes were blocked with 5% skimmed milk at 5 °C for 1 h. The primary antibody was diluted with 5% BSA at 4 °C and incubated with the membranes overnight at 4 °C. Rabbit anti-human NR4A1 polyclonal antibody (1:1000, cat. no. orb127604, Biorbyt), rabbit anti-human NF-κB p-p65 polyclonal antibody (1:100, cat. no. orb501839, Biorbyt,), rabbit anti-human NF-Κb p65 polyclonal antibody (1:100, cat. no. orb344389, Biorbyt), rabbit anti-human IκBα polyclonal antibody (1:1000, cat. no. 4812, Cell Signalling Technology, Inc.), rabbit anti-human p-IκBα polyclonal antibody (1:800, cat. no. 2859, Cell Signalling Technology, Inc.) and rabbit anti-human GAPDH polyclonal antibody (1:1000, cat. no. 5174, Cell Signalling Technology, Inc.) were sequentially added to the specimens. Then, the samples were rinsed with 0.01% TBST every 3 times for 10 min, combined with horseradish peroxidase-conjugated goat anti-rabbit IgG secondary antibody (1:1000, cat. no. K0034G-AF594, Beijing Solarbio Science & Technology Co., Ltd.) and incubated at room temperature for 1 h. TBST was used to wash the specimens three times. The protein bands were observed by ECL chemiluminescence reagent (cat. no. GE2301, GenView Scientific) and the protein expression levels were calculated by ImageJ software (version 6; National Institutes of Health).

### Molecular docking analysis

The PTE ligand was retrieved from PubChem (https://pubchem.ncbi.nlm.nih.gov/) with 3D structure, and the three-dimensional structure of NR4A1 was downloaded from PDB database (https://www.rcsb.org/). Mechanical optimization, hydrogenation, and charging of ligand were carried out by UCSF chimaera software (https://www.cgl.ucsf.edu/chimaera/). Then the molecular docking was analyzed using the AutoDock Vina tool. A grid box was generated that was large enough to cover the entire protein binding site and allow all ligands to move freely (Tippani et al. 2014). The docking results were evaluated using a total-score.

### Statistical analysis

SPSS 19.0 software (SPSS Inc.) was used to conduct data analysis. One-way ANOVA was performed for multiple comparisons, followed by Tukey’s *post-hoc* test. Data are presented as mean ± SD. *p* < 0.05 was considered to indicate statistically significant differences.

## Results

### Protective effect of PTE on LPS induced ALI

**Figure 2.**
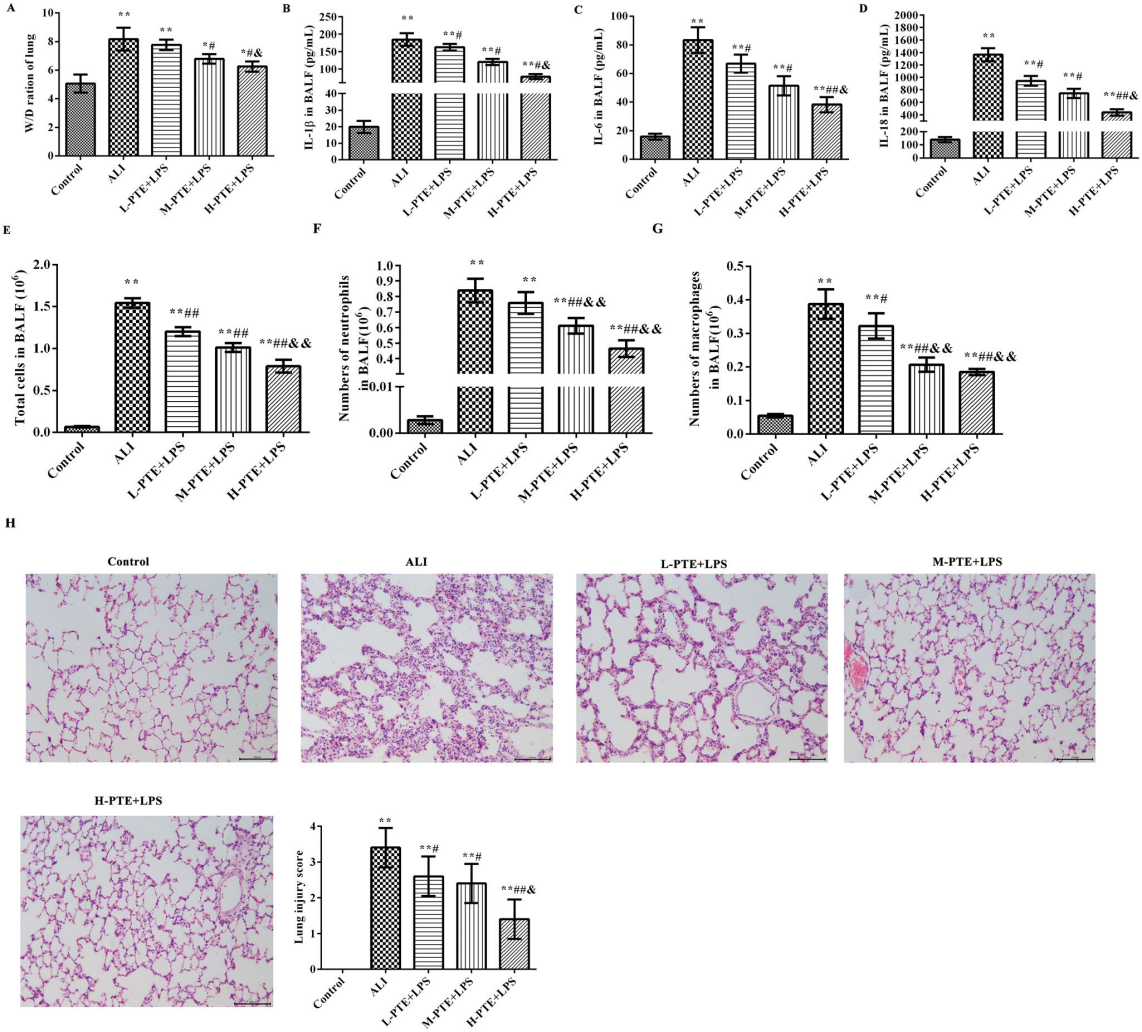
Effect of PTE pre-treatment on LPS induced ALI rats. (A) Lung W/D ratio. (B) The levels of IL-1β in the BALF were analyzed by ELISA; (C) the levels of IL-6 in BALFs were analyzed by ELISA; (D) the levels of IL-8 in BALF were analyzed by ELISA; (E) the numbers of total cells in BALF; (F) the numbers of neutrophils in BALF; (G) the numbers of macrophages in BALF; (H) the lung pathology was observed by haematoxylin-eosin (HE) staining (scale = 100 μm). *p < 0.05, **p < 0.01 *vs.* control group. ^#^*p* < 0.05, ^##^*p* < 0.01 *vs.* ALI group. ^&^*p* < 0.05, ^&&^*p* < 0.01 *vs.* L-PTE + LPS group. L-PTE: low dose of PTE (10 mg/kg); M-PTE: medium dose of PTE (20 mg/kg); H-PTE: high dose of PTE (40 mg/kg); BALF: bronchoalveolar lavage fluid; ALI: acute lung injury; W/D: wet/dry.

As shown in Figure 2(A), the lung W/D ratio was significantly increased in the ALI group compared with that in the control group (*p* < 0.01). The lung W/D ratio decreased after pre-treatment with PTE in a dose-dependent manner. Specifically, the lung W/D ratio was significantly decreased in the H-PTE + LPS group compared with that in the L-PTE + LPS group (*p* < 0.05).

The expression of the pro-inflammatory factors IL-1β, IL-6, and IL-18 was also examined (Figure 2(B–D)). The results revealed that the expression levels of these factors in the ALI group were significantly higher compared with those in the control group (*p* < 0.01). However, the levels of these factors were significantly reduced after pre-treatment with PTE in a dose-dependent manner (*p* < 0.05). Specifically, these expression levels were significantly reduced in the H-PTE + LPS group compared with those in the L-PTE + LPS group (*p* < 0.05).

Additionally, the numbers of total cells (Figure 2(E)), neutrophils (Figure 2(F)), and macrophages (Figure 2(G)) in BALFs of each group were counted. Compared with the control group, the numbers of total cells, neutrophils and macrophages were significantly increased in the LPS group (*p* < 0.01). With the doses of PTE increasing, the numbers of these inflammatory cells were decreased.

The effects of pre-treatment PTE were further investigated by observing the pathological changes of the lung tissue (Figure 2(H)). It was observed that pre-treatment PTE significantly reduced the infiltration by inflammatory cells and the lung injury score.

### Effect of pre-treatment PTE on NR4A1 expression in LPS induced ALI

**Figure 3.**
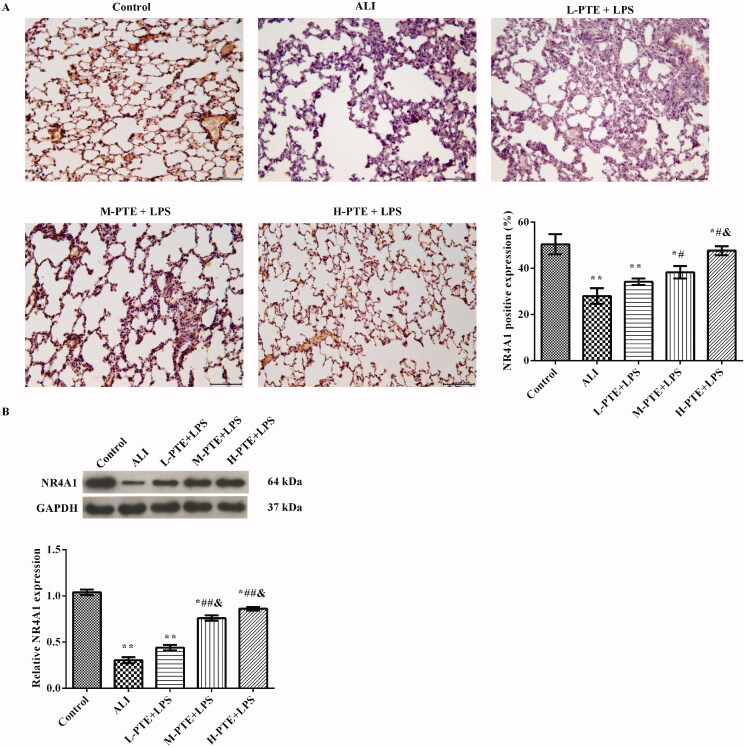
Effect of PTE pre-treatment on NR4A1 expression in LPS induced ALI rats. The expression of NR4A1 was analyzed by (A) immunohistochemistry (scale = 100 μm) and (B) western blotting in the lung tissues of each group. *p < 0.05, **p < 0.01 *vs.* control group. ^#^*p* < 0.05, ^##^*p* < 0.01 *vs.* ALI group. ^&^*p* < 0.05 *vs.* L-PTE + LPS group. L-PTE, low dose of PTE (10 mg/kg); M-PTE, medium dose of PTE (20 mg/kg); H-PTE, high dose of PTE (40 mg/kg); ALI, acute lung injury.

Immunohistochemistry (Figure 3(A)) and western blot (Figure 3(B)) were used to analyze the expression of NR4A1 in lung tissues. We found that the expression of NR4A1 was significantly decreased in the ALI group when compared to the control group (*p* < 0.05). The expression of NR4A1 was increased significantly after being pre-treated with PTE. There was a more obvious increasing tendency with the high concentration (*p* < 0.05).

### Effect of pre-treatment PTE on the NF-κB pathway in LPS induced ALI

**Figure 4.**
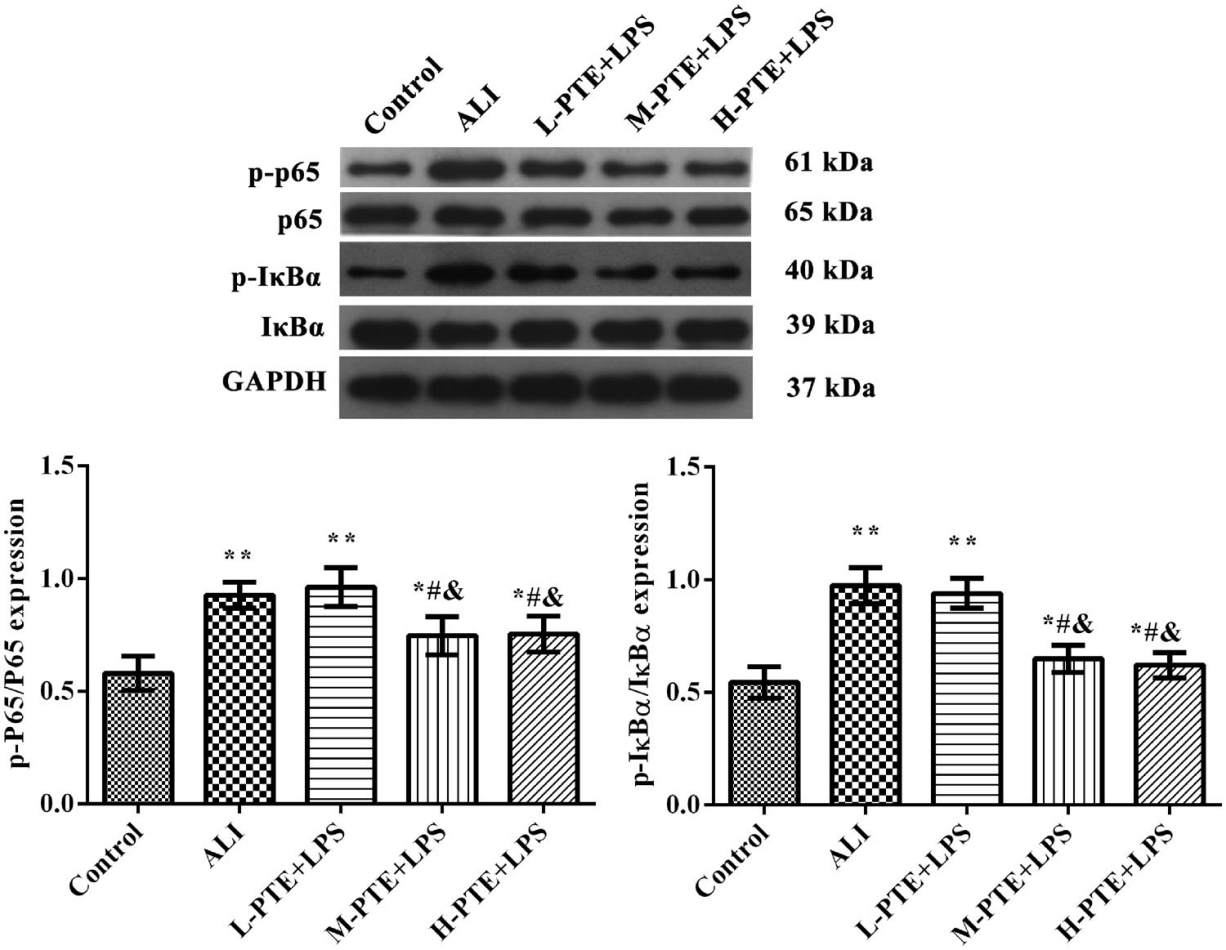
Western blotting was used to detect the protein levels of p-p65, p65, p-IκBa, and IκBa in lung tissue. GAPDH was used as an internal control. *p < 0.05, **p < 0.01 *vs.* control group. ^#^*p* < 0.05, *p* < 0.01 *vs.* ALI group. ^&^*p* < 0.05 *vs.* L-PTE + LPS group. L-PTE: low dose of PTE (10 mg/kg); M-PTE: medium dose of PTE (20 mg/kg); H-PTE: high dose of PTE (40 mg/kg); ALI: acute lung injury.

To further elucidate the mechanisms underlying the protective effects of pre-treatment PTE, western blotting was used to analyze the levels of NF-κB pathway-related proteins in lung tissue. As shown in Figure 4, the protein levels of p-p65/p65 and p-IκBa/IκBa were notably increased in the ALI group compared with those in the control group (*p* < 0.01). After pre-treatment with PTE, the expression of p-p65/p65 and p-IκBa/IκBa was markedly decreased (*p* < 0.05). Moreover, the protein levels of NF-κB pathway-related proteins in the H-PTE + LPS group were markedly higher compared with those in the L-PTE + LPS group (*p* < 0.05).

### Pre-treatment PTE exerts a protective effect against LPS induced ALI through NR4A1

**Figure 5.**
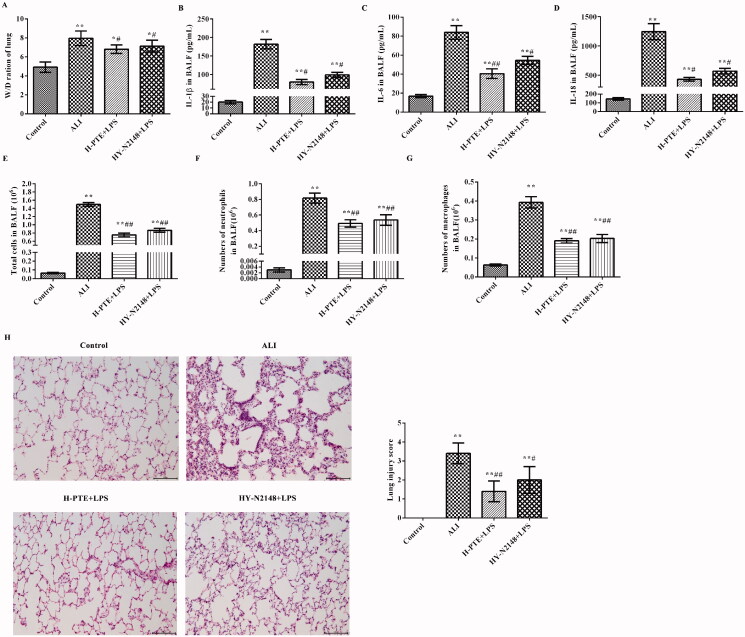
PTE pre-treatment exerts a protective effect against LPS induced ALI through NR4A1. (A) Lung W/D ratio in each group; (B–D) ELISA was used to detect the expression of IL-1β, IL-6, IL-8 in the BALFs. (E) the numbers of total cells in BALFs; (F) the numbers of neutrophils in BALFs; (G) the numbers of macrophages in BALFs; (H) HE staining was used to analyze the pathological changes of the lung tissue (scale = 100 μm). *p < 0.05, **p < 0.01 *vs.* control group. ^#^*p* < 0.05, ^##^*p* < 0.01 *vs.* ALI group. H-PTE: high dose of PTE (40 mg/kg); HY-N2148: NR4A1 agonist (Cytosporone B) (50 μl, 1 μM); BALFs: bronchoalveolar lavage fluids; ALI: acute lung injury; W/D: wet/dry.

As shown in Figure 5(A), the lung W/D ratios in the H-PTE + LPS and HY-N2148 + LPS groups were significantly lower compared with those in the ALI group (*p* < 0.05). The results from serial analysis of inflammatory factors in the BALFs demonstrated that the levels of IL-1β, IL-6, and IL-18 in the ALI group were significantly higher compared with those in the other groups (*p* < 0.05; Figure 5(B–D)). After pre-treatment with high-dose PTE or NR4A1 agonist, the levels of the above inflammatory factors were significantly reduced (*p* < 0.05). In addition, the numbers of total cells (Figure 5(E)), neutrophils (Figure 5(F)), and macrophages (Figure 5(G)) in BALFs were clearly decreased in HY-N2148 + LPS group compared with the ALI group (*p* < 0.01). After further observing the pathological changes in the lung tissues of each group, it was observed that high-dose PTE or NR4A1 agonist significantly reduced the inflammatory cell infiltration and the lung injury score (*p* < 0.05; Figure 5(E)).

### Pre-treatment PTE regulates the NF-κB pathway in LPS induced ALI through NR4A1

**Figure 6.**
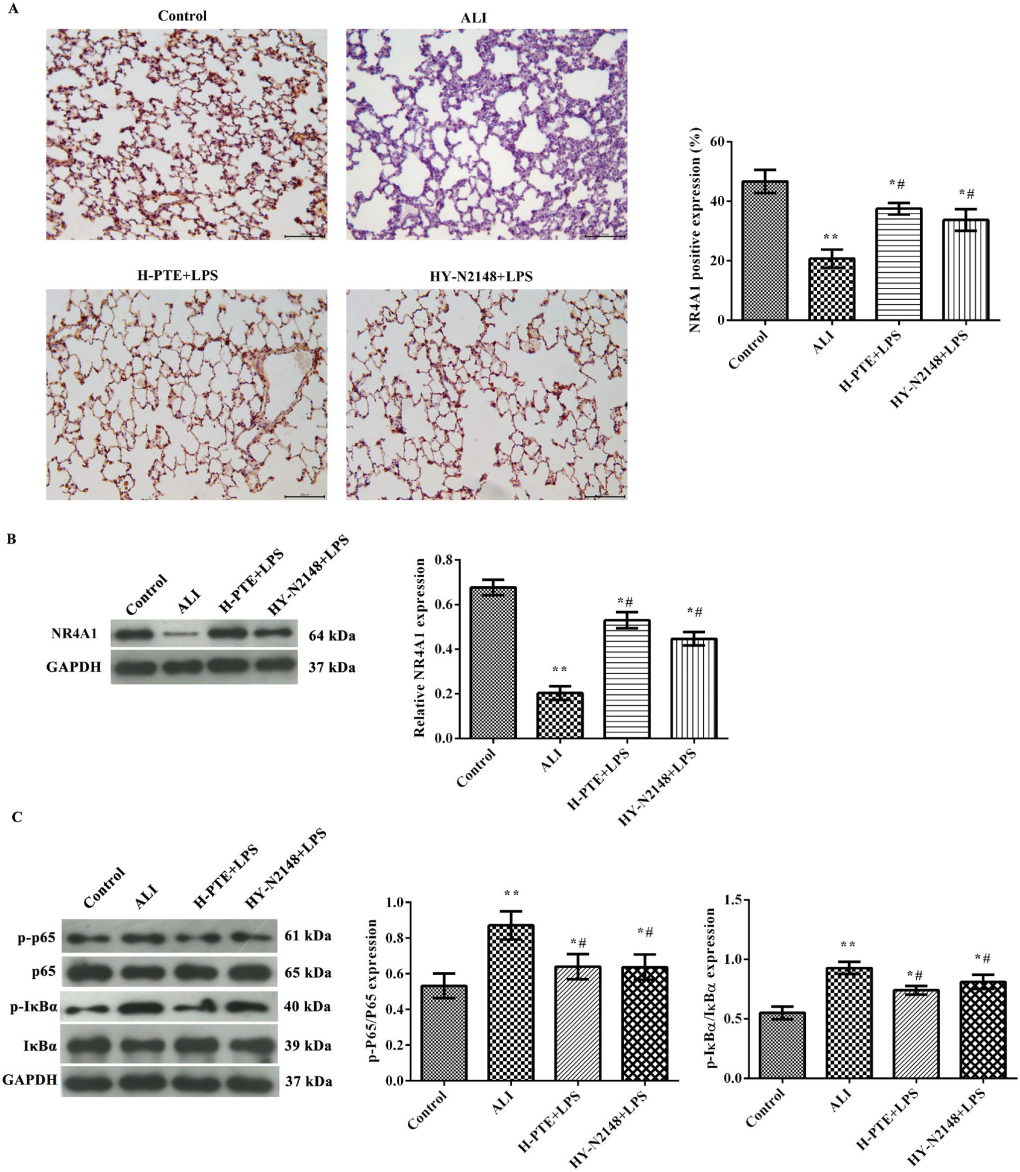
PTE pre-treatment regulates the NF-κB pathway in LPS induced ALI through NR4A1. The expression of NR4A1 in lung tissues was examined by (A) immunohistochemistry (scale = 100 μm) and (B) western blotting. (C) The expression of p-p65, p65, p-IκBα, and IκBα was analyzed by western blotting. GAPDH was used as an internal control. *p < 0.05, **p < 0.01 *vs.* control group. ^#^*p* < 0.05 *vs.* ALI group. H-PTE: high dose of PTE (40 mg/kg); HY-N2148: NR4A1 agonist (Cytosporone B, 50 μL, 1 μM); NF-κB: nuclear factor-κB; ALI: acute lung injury.

Immunohistochemistry (Figure 6(A)) and western blotting (Figure 6(B)) were employed to analyze NR4A1 expression in the lung tissues of each group. It was observed that, compared with the ALI group, the NR4A1 expression was significantly increased following pre-treatment with PTE or NR4A1 agonist (*p* < 0.05). To further investigate the association among PTE, the NF-κB pathway and NR4A1, the p-p65/p65 and p-IκBα/IκBα protein levels were examined. The results demonstrated that compared with the ALI group, the protein levels of p-p65/p65 and p-IκBα/IκBα were markedly reduced following pre-treatment with PTE or NR4A1 agonist (*p* < 0.05; Figure 6(C)).

### A good activity of PTE to NR4A1 and pre-treatment of PTE significantly prevents the death of LPS-treated rats

**Figure 7.**
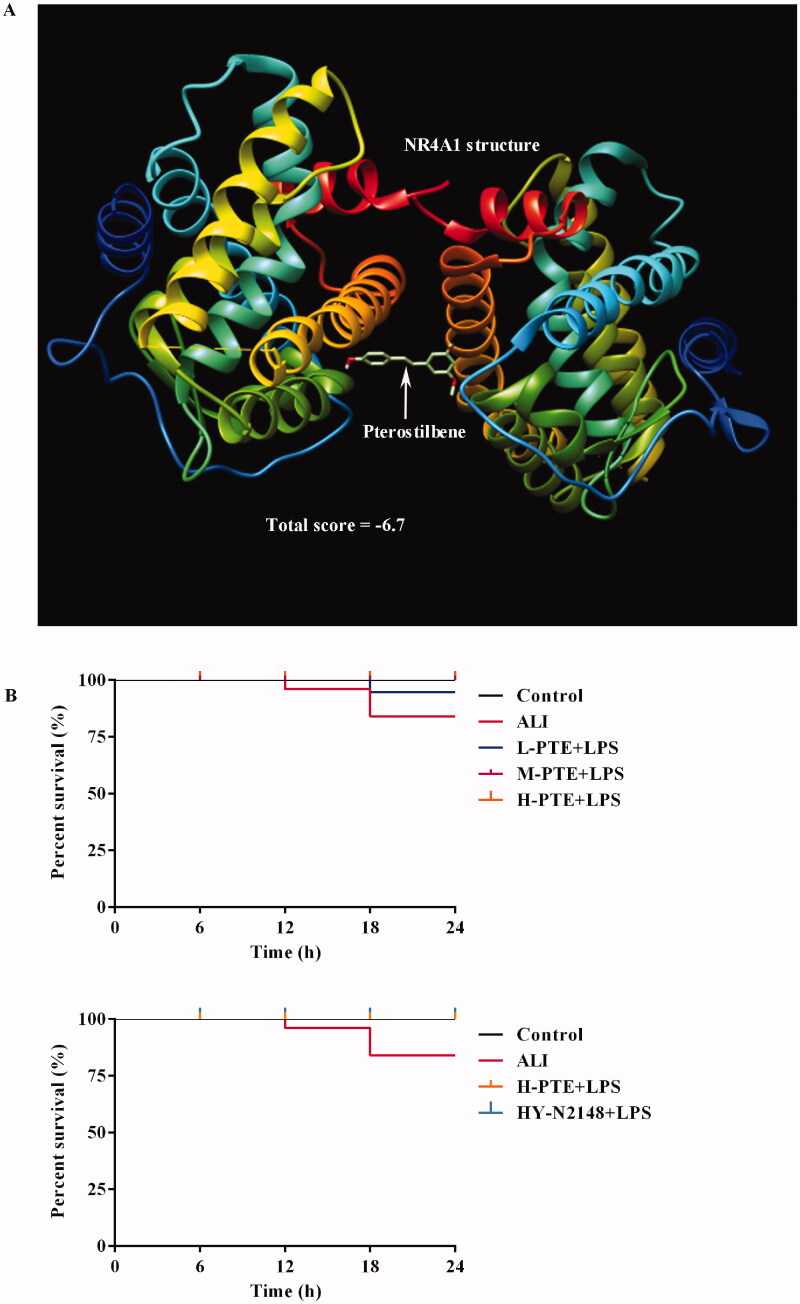
The binding activity of PTE to NR4A1, and survival rate in this study. (A) The binding activity of PTE to NR4A1 was forecasted using UCSF chimaera and AutoDock Vina softwares. There is a good binding activity of PTE to NR4A1 (total score = −6.7). (B) The survival rate was tracked every 6 h after the LPS instillation. The percent survival was expressed as a Kaplan-Meier survival curve.

The total score means inter molecular energy (kcal/mol), which represents the stability between the ligand and receptor. The value is more negative, the binding is more stable. Taking the absolute value of a total score >6 as the screening condition, the molecular docking results showed that there was a good activity of PTE to NR4A1 (total score = −6.7, Figure 7(A)). The survival rate was 70% after LPS treatment 24 h in the ALI group, but the PTE pre-treatment before LPS administration prevented the death of rats (Figure 7(B)). In the L-PTE + LPS group, the survival rate was 90% after LPS treatment 24 h, and the survival rate was 100% in the M-PTE + LPS group and H-PTE + LPS group. Similarly, the survival rate was 100% in the HY-N2148 + LPS group.

## Discussion

Several beneficial biological and pharmacological effects of PTE have been demonstrated in arthritis (Perecko et al. 2013), acute seizures (Nieoczym et al. 2019), and diabetic complications (Dodda et al. 2020). Moreover, PTE may be of value in cancer chemoprevention based on its strong anti-NF-κB and anti-inflammatory properties (Nikhil et al. 2015). The anti-inflammatory and antioxidative activities of PTE for alleviating renal and liver fibrosis were recently reported (Liu et al. 2019). According to a previous report (Du et al. 2018), the lung W/D ratio is commonly increased in LPS-induced lung damage due to the increased pulmonary vascular permeability to water. Consistent with these reports, the present study also demonstrated that PTE pre-treatment significantly decreased the lung W/D ratio, decreased inflammatory cells (total cells, neutrophils, and macrophages), and inflammatory factors (IL-6, IL-1β, and IL-18) in BALFs and improved the lung pathology in LPS-induced ALI rats. These results suggested that PTE was a potential preventive drug for ALI caused by LPS. In addition, the expression of NR4A1 was increased significantly after pre-treatment with PTE, and this increase was concentration-dependent. These findings indicated that NR4A1 might represent an appropriate therapeutic target of PTE for preventing ALI induced by LPS.

In the immune system, previous studies knocked out NR4A1 in animal models to investigate its role in the regulation of various immune responses (Duren et al. 2016; Cui et al. 2019). NF-κB signalling is a major driver of inflammatory responses through promoting the expression of pro-inflammatory mediators, such as chemokines and adhesion molecules (Banno et al. 2019). Specifically, pre-treatment with the NR4A1 agonist (Cytosporone B) blocked the endothelin-1 expression in the lungs of LPS-exposed rats, and this suppression was mediated through NF-κB signalling (Jiang et al. 2016). The present study also demonstrated that NR4A1 exerted a beneficial effect by suppressing the expression of p-p65/p65 and p-IκBα/IκBα in lung tissues of LPS caused ALI rats by pre-treatment with the NR4A1 agonist (Cytosporone B). Of note, the effect of PTE pre-treatment was similar to that of Cytosporone B pre-treatment. These data indicated that PTE pre-treatment might play a protective role in LPS-induced ALI by activating NR4A1 to regulate the NF-κB pathway.

To confirm whether PTE directly binds to NR4A1, we used molecular docking analysis to forecast the binding activity. The total score showed there was a good binding activity of PTE to NR4A1. It is widely known that PTE directly binds to the crystal structure of telomerase (3DU6) to inhibit the cell viability in breast and lung cancer (Tippani et al. 2014). In addition, Bhakkiyalakshmi et al. (2016) revealed that PTE directly interacted with the basic amino acids of the kelch domain of Keap1 and perturb Keap1-Nrf2 interaction pattern using molecular docking and dynamic simulation. For developing a novel anti-*Staphylococcus aureus* (MRSA), the structure of PTE was used as a scaffold for the hybrid 1,2,3-triazole moiety (Tang et al. 2019). In our study, the survival rate of LPS treated rats was also increased because of PTE pre-treatment. These data indicated that PTE might be a better potential drug with fewer side effects, as a natural bioactive compound.

However, this study focussed on PTE administration before the LPS challenge, which was different from clinical practice. The effects of PTE by administrating it after the onset of LPS-induced ALI are needed to be studied, and determination of the roles of PTE and NR4A1 in the pathophysiology of ALI are necessary. Furthermore, the mechanisms of PTE on other inflammatory factors requires further investigation in the ALI.

## Conclusions

In the present study, PTE pre-treatment significantly decreased the inflammatory responses in the LPS-induced ALI rats. In addition, the effects of pre-treatment with the NR4A1 agonist (Cytosporone B) were similar to PTE pre-treatment. These data suggest PTE pre-treatment may exert a protective effect against LPS-induced ALI by activating NR4A1.
